# The Potential Influence of Bumble Bee Visitation on Foraging Behaviors and Assemblages of Honey Bees on Squash Flowers in Highland Agricultural Ecosystems

**DOI:** 10.1371/journal.pone.0144590

**Published:** 2016-01-14

**Authors:** Zhenghua Xie, Dongdong Pan, Jonathan Teichroew, Jiandong An

**Affiliations:** 1 Department of Environmental Entomology, Research Institute of Insect Resources, Chinese Academy of Forestry, Kunming, Yunnan, China; 2 Department of Statistics, Yunnan University, Kunming, Yunnan, China; 3 Key Laboratory of Economic Plants and Biotechnology, Kunming Institute of Botany, Chinese Academy of Science, Kunming, Yunnan, China; 4 University of Chinese Academy of Sciences, Beijing, China; 5 Institute of Apicultural Research, Chinese Academy of Agricultural Sciences, Beijing, China; The National Orchid Conservation Center of China; The Orchid Conservation & Research Center of Shenzhen, CHINA

## Abstract

Bee species interactions can benefit plant pollination through synergistic effects and complementary effects, or can be of detriment to plant pollination through competition effects by reducing visitation by effective pollinators. Since specific bee interactions influence the foraging performance of bees on flowers, they also act as drivers to regulate the assemblage of flower visitors. We selected squash (*Cucurbita pepo* L.) and its pollinators as a model system to study the foraging response of honey bees to the occurrence of bumble bees at two types of sites surrounded by a high amount of natural habitats (≥ 58% of land cover) and a low amount of natural habitats (≤ 12% of land cover) in a highland agricultural ecosystem in China. At the individual level, we measured the elapsed time from the departure of prior pollinator(s) to the arrival of another pollinator, the selection of honey bees for flowers occupied by bumble bees, and the length of time used by honey bees to explore floral resources at the two types of sites. At the community level, we explored the effect of bumble bee visitation on the distribution patterns of honey bees on squash flowers. Conclusively, bumble bee visitation caused an increase in elapsed time before flowers were visited again by a honey bee, a behavioral avoidance by a newly-arriving honey bee to select flowers occupied by bumble bees, and a shortened length of time the honey bee takes to examine and collect floral resources. The number of overall bumble bees on squash flowers was the most important factor explaining the difference in the distribution patterns of honey bees at the community level. Furthermore, decline in the number of overall bumble bees on the squash flowers resulted in an increase in the number of overall honey bees. Therefore, our study suggests that bee interactions provide an opportunity to enhance the resilience of ecosystem pollination services against the decline in pollinator diversity.

## Introduction

Wild bees and managed honey bees are declining at both local and global scales [1–3]. Habitat loss is one of the key factors driving the decline of wild bees. The responses of wild bees and managed honey bees to habitat loss are often quite different due to their diverse life history [4], and are affected differently according to the intensity of environmental disturbances [5]. In many cases, when wild bee diversity declines due to anthropogenic disturbances, honey bee abundance does not respond in kind [4–6]. While the spatial-temporal patterns of visiting densities of wild bees and honey bees in agricultural ecosystems have been well documented [5], the species interactions between wild bees and honey bees in degraded habitats are not well understood.

Inter- and intra-specific bee species interactions act as important factors regulating the foraging activities of bees on flowers and the services they provide to plants. Conspecific and heterospecific encounters benefit crop pollination through several mechanisms known as synergistic effects [3,7,8] or complementary effects [9,10]. On the contrary, bee interactions are recognized as drivers to repel effective pollinators [11,12], showing negative effects on plant pollination. Numerous studies have revealed the negative influence of alien pollinators, such as *Apis mellifera* and *Bombus terrestris*, on the restricted foraging activities of native pollinators [13,14], leading to pollinator replacement and reduced pollination [14,15]. However, the functions of bee interactions are not fully understood. Since bee interactions show diverse effects on pollinator performance [12,16], they can influence the flower visitors and result in different pollination services to plants [17]. Therefore, bee interactions may act as drivers regulating pollinator assemblages and pollination services of ecosystems.

In this study, we selected the squash flower (*Cucurbita pepo* L.) and its pollinators as a model system in the highland agricultural ecosystems in China to explore the effects of species interactions between bumble bees and honey bees on the foraging activities and assemblages of honey bees. Bumble bees and Asian honey bees (*Apis cerana* Fab.) are native pollinators of squash in this region [18]. At the individual level, we explored the variability of foraging activities of honey bees in response to the occurrence of bumble bees on squash flowers. At the community level, we studied the assemblages of honey bees in response to the occurrence of bumble bees. Specifically, we examined the following four questions: 1. Do flowers previously visited by a bumble bee experience a longer period of time before it is revisited by another newly-arriving bee compared to the flowers previously visited by a honey bee? 2. What are the selections of flowers by honey bees which have been previously occupied by bumble bees? 3. Do honey bees take an increasing amount of time to collect food resources from squash flowers when bumble bees are present and visiting the flowers? 4. How do bumble bees influence the distribution patterns of honey bees on squash at a community level?

## Materials and Methods

### Ethics statement

This study was carried out in private, farm-owned fields, and all farmers gave permission to conduct the study. No additional permits or approvals were needed to sample the bees because the fields are not protected in any way. The field studies did not involve endangered or protected species.

### Study site, crop and pollinator

The study was carried out at eight sites near Kunming (25°07’N, 102°50’E), Yunnan Province, China. The study sites were characterized by two different land-use types, with four sites surrounded by a high proportion (≥ 58%) of natural and semi-natural habitats (forest and grassland) (hereafter HN habitats) and another four sites by a low proportion (≤ 12%) of natural and semi-natural habitats (hereafter LN habitats) (Table 1). Previous studies have demonstrated that natural and semi-natural habitats surrounding agricultural ecosystems determined bumble bee visitation rates to squash flowers and thereby secure crop pollination in the highland agricultural ecosystems [18]. Only honey bees were observed on squash flowers at the four sites in the LN habitats according to our field observations for five years (2009–2014). Managed Asian honey bees, *A*. *cerana*, were abundant at both types of sites [18]. Our interviews with local farmers and residents indicated that at least five colonies of managed honey bees were set within a distance of 500 m to the fields, ensuring all fields had large honey bee populations nearby. As a result, the four sites in the HN habitats had both bumble bees and honey bees present, whereas the other four sites in the LN habitats had only honey bees. The minimum distance between each of the sites was 2.0 km, which was longer than the general foraging distance of bumble bees [19,20] and Asian honey bees [21]. Wild flowering plants around the fields included *Trifolium* spp., *Vicia* spp., *Rosa* spp., *Hypericum bellum* Li, and *Oenothera rosea* L. The small populations of wild flowers were irregularly located at the field margins, hedgerows, and surrounding natural habitats. Areas of squash fields ranged from 221 m^2^ to 340 m^2^ (mean = 257), whereas wild plants were often in small patches and covered several square meters per occurrence, suggesting that the squash flowers at the peak of blooming produced a higher amount of floral resources (nectar and pollen) than the wild plants. There was no other massive flower crop at the two types of fields during the field surveys. Pesticides and herbicides were applied at all field sites, but field surveys were conducted at least two days after chemical applications.

**Table 1:** The eight study sites exploring the influences of bumble bee visitation on the foraging behaviors and distribution patterns of honey bees.

			Effects at the individual level ^b^			Effects at the community level ^b^	
Sites	Percentage of natural and semi-natural habitat ^a^	Occurrence of bumble bee visitation on squash flowers	Period for Departure-arrival Route	Period for Behavioral Encounters on Flower Petals	Period for Examining and Collecting Food Resources	Foraging patterns of honey bee community	No. of fields
**Zhuangfan**	0.65	yes		√	√	√	8
**Tuanshan**	0.58	yes				√	7
**Dabai**	0.62	yes	√	√	√	√	2
**Sansimu**	0.69	yes		√	√	√	2
**Dabanqiao**	0.02	no		√	√	√	5
**Shuanglong**	0.12	no	√	√	√	√	6
**Shujie**	0.09	no		√	√	√	3
**Changkou**	0.02	no				√	2

^a^ Percentage of natural and semi-natural habitats (forest and grassland) was calculated at 750 m spatial scale.

^b^ √ indicates the sites where field sampling was carried out.

*Cucurbita pepo* had separate pistillate or staminate flowers, both of which produced a high volume of nectar, ranging from 90 to 120 *u*l for pistillate flowers and 25 to 40 *u*l for staminate flowers [22,23]. Additionally, the staminate flowers produced an average of 16 × 10^3^ pollen grains per flower [23]. Pollinators directly imbibed the nectar distribution around the base of the style bordered by an annulus [23]. On the staminate flowers, pollinators collected the nectar through nectary pores at the base of staminate flowers by extending their proboscises to the underneath the antheriferous columns (anthers) [23]. All field observations and sampling were conducted at peak flowering time, on sunny days with wind speeds less than 0.3 m/s.

### Effects on foraging behaviors of honey bees at the individual level

**Fig 1:**
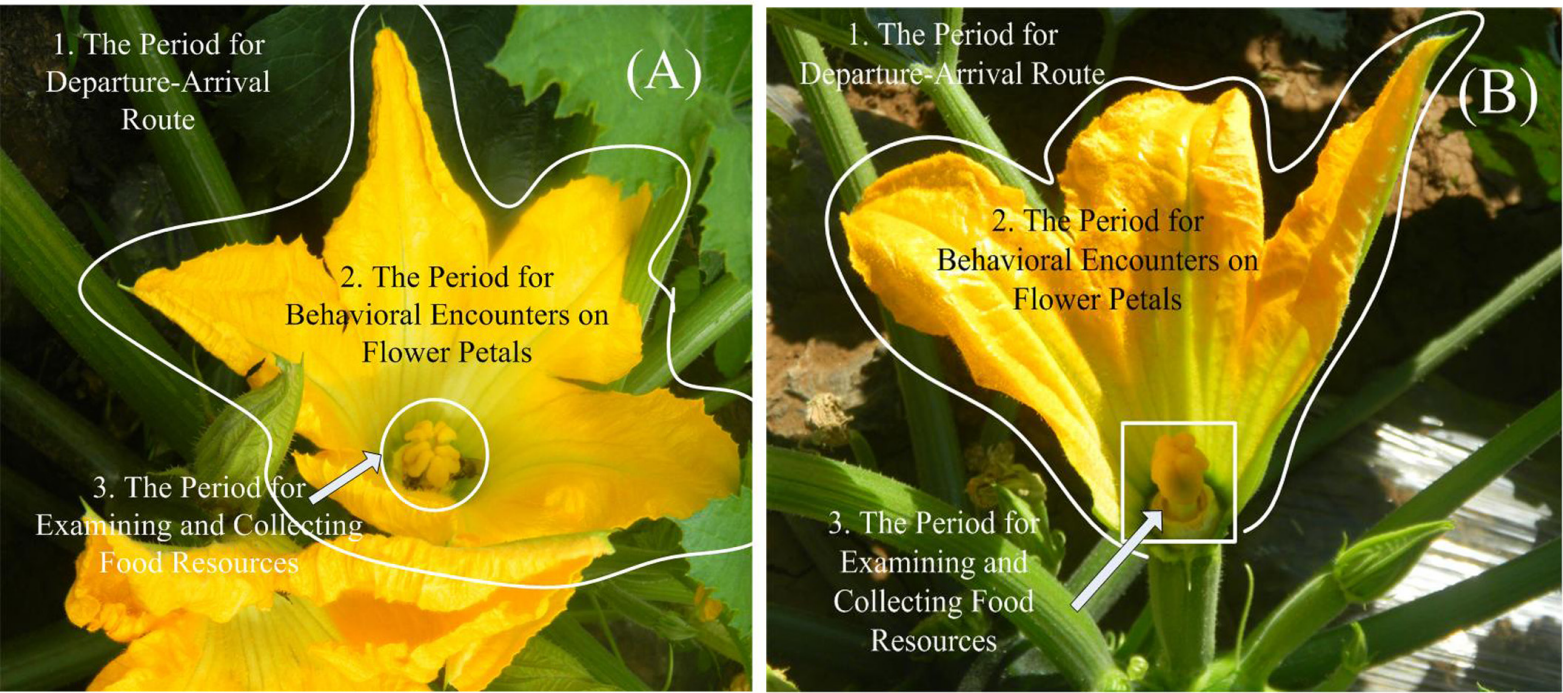
Three periods of foraging behavior of honey bees on a squash flower. (A) top view; (B) lateral view.

Foraging behavior of pollinators collecting floral resources on squash flowers was arbitrarily divided into three successive periods, based on both the bees’ positions on squash flowers (e.g. pre-arrival at the flowers; landing on the petal of the flowers; and lingering near the stigma/anther of the flowers) and the differences in which pollinators interacted with other individuals. The three periods were named as: the Period for Departure-arrival Route, the Period for Behavioral Encounters on Flower Petals, and the Period for Examining and Collecting Food Resources (Fig 1).

The Period for Departure-arrival Route referred to the duration lasting from the departure of prior bee(s) to the arrival of conspecifics or heterospecifics. It was measured using the elapsed time (in seconds) from the departure of the prior pollinator(s) to the arrival of another pollinator, also formulated here as a departure-arrival route. During this period, pollinators mainly use olfactory cues to locate the flowers, without any physical contact with other individuals. We hypothesized that the flowers previously visited by bumble bees required a longer time to be revisited by a newly-arriving honey bee, compared to the flowers last visited by honey bees (Fig 1).

The Period for Behavioral Encounters on Flower Petals referred to the duration from the bee landing on a petal surface to its arrival at the base of the stigma/anther. It was measured using the behavioral response (e.g. probing or leaving) of the newly-arrived pollinators to the flowers occupied by other conspecifics or heterospecifics. During this period, pollinators mainly used visual and tactile cues, potentially with physical contacts with other individuals. We expected that honey bees preferred leaving the flower after encountering bumble bees (Fig 1).

The Period for Examining and Collecting Food Resources referred to the duration from bee arrival to the base of stigma/anther to departure always from the flower, and this period potentially contained behaviors such as examining and collecting nectar and pollen. We measured using the length of time (in seconds) for pollinators to examine and collect the floral resources. During this period, pollinators had physical contact with other conspecifics or heterospecifics. The length of time was associated with the amount of floral resources. Honey bees were assumed to spend more time in handling floral resources at sites within the LN habitats than at sites within the HN habitats (Fig 1).

Differences in foraging activities of the pollinators during the three periods were compared between the HN and LN habitats. Differences in bee interactions across the three periods (a full analysis of foraging behavior on a squash flower) could be revealed by the aggregate effects from the three individual periods we measured.

#### The period for departure-arrival route

We conducted sampling in two fields, with one field within the HN habitats (called Dabai) and another field within the LN habitats (called Shuanglong) (Table 1). One 20 m × 2 m transect was set in the center of each field; five 2 m × 2 m sample plots separating each other with a distance of 2 m were placed within the transect. One pistillate flower and one staminate flower from each plot were randomly marked and all observations were conducted on the flowers. In total, five pistillate flowers and five staminate flowers were selected from each field. The observers stood outside the sample plots and recorded all bees (both honey bees and bumble bees) subsequently visiting the flower. Using a stopwatch, we measured the elapsed time (in seconds) from the departure of the prior bee(s) until the arrival of the next bee landing on the petal of the flower. Bees were recorded as honey bees or bumble bees in fields because we wanted to know how honey bees and bumble bees influenced each other. If two or more honey bees were simultaneously occupying the flower during the field sampling, which occurred in Shuanglong, the elapsed time was recorded from the departure of last bee. We did not find any two individuals (either two bumble bees or two honey bees) simultaneously foraging on a single flower at Dabai. The two flowers within the sample plot, as well as the plots within the transect, were sampled successively, with each flower sampled for at least 20 minutes. This methodology was useful to measure the length of time for a departure-arrival route with known prior bee(s) and the next-arriving bee in field conditions. Field sampling was conducted between 08:00 am to 10:30 am from May 10 to June 7, 2014. A total of 300 min were sampled at Dabai and 280 min at Shuanglong, which included 187 and 120 departure-arrival routes, respectively.

At Dabai, the elapsed time sampled on pistillate and staminate flowers were analyzed separately due to their differences in rewards and attractiveness to bees [22,23], using student *t* test. At Shuanglong, the sample size of the elapsed time on staminate flowers was quite small (n = 13). Moreover, the elapsed time was just recorded for staminate flowers previously occupied by only one honey bee. We compared the elapsed time for the pistillate flowers and the staminate flowers both previously occupied by only one honey bee and found there was no significant difference between them (*t* test: *t* = 0.304, df = 15.34, *p* = 0.765; log (x+1) transformation of variables). Therefore, the elapsed time for pistillate and staminate flowers both previously occupied by one honey bee was pooled together. One-way Analysis of Variance (ANOVA) was conducted to test the differences in the elapsed time for the flowers previously occupied by one, two, three, or four honey bees. The elapsed time was log (x+1) transformed to meet the assumption of potential normally distributed residuals and homogeneity of variance. Student *t* test and ANOVA were conducted using R 3.1.0 software [24].

#### The period for behavioral encounters on flower petals

Field sampling was carried out in six fields, with three fields within the HN habitats and three fields within the LN habitats (Table 1). One 20 m × 2 m transect was placed in the center of each field. The observers walked along the center line of transect and selected the flowers occupied by bees (e.g. bumble bees or honey bees) collecting floral resources. When a newly-arriving bee landed on the petal, we recorded the response of the newly-arriving bee as ‘Probing’ if it walked towards stigma/anther to examine food resources; otherwise, we recorded the response of the newly-arrived bee as ‘Leaving’ if it flew away within two seconds. The newly-arrived bee and the bee(s) already occupying the flower were recorded as bumble bees or honey bees. The foraging responses were not used if two bees simultaneously arrived and landed on the petal of the same flower, because we were not interested in exploring the responses of one newly-arrived bee to another newly-arrived bee. Each transect was sampled four times to achieve a large sample size between 07:30 am– 11:30 am from May 15 to June 7, 2013, and May 7 to June 16, 2014. The transect was sampled once in 2013 and re-sampled three additional times in 2014. In total, 126 instances of behavioral encounters were engaged at the three fields within the HN habitats and 205 instances at the three fields with the LN habitats.

Four types of encounters (one honey bee encountering one bumble bee, one honey bee encountering another honey bee, one bumble bee encountering another bumble bee, and one bumble bee encountering one honey bee, see Results) were observed across the three fields within the HN habitats. Since sample sizes per-field and per-year were small and insufficient to test individually, each type of the four encounters from different years and fields were pooled, respectively. Meanwhile, three types of encounters (one honey bee encountering another honey bee, one honey bee encountering two honey bees, and one honey bee encountering three honey bees, see Results) were sampled across the three fields within the LN habitats. Each type of the three encounters from different years and fields were also pooled due to a small sample size, respectively. The chi-square goodness-of-fit test was applied to test whether the bees had an equal probability in flower selection (leaving or probing).

#### The period for examining and collecting food resources

Field sampling was carried out in six fields, with three fields within the HN habitats and three fields within the LN habitats (Table 1). One 20 m × 2 m transect was placed in the center of fields. The observers walked along the center line of the transect and selected the flowers unoccupied by any bees. If a newly-arriving bee (e.g. honey bee or bumble bee) happened to land on the flowers and arrived near the base of the stigma/anther, the observers recorded the time, using a stopwatch, from the arrival until the departure of the bee(s). The flowers were abandoned if two pollinators simultaneously accessed the same flower, because the two newly-arrived bees likely disturbed each other and reduced the length of time used to examine and collect floral resources. Each transect was sampled once with a similar speed (approximately 10 minutes). Fields were sampled on sunny days, with favorable ambient temperatures (18–22°C). Field sampling was conducted from May 20 to June 10, 2014.

The length of time was analyzed separately for pistillate and staminate flowers due to their differences in rewards and attractiveness to visitors. Since sample sizes of fields between the HN habitats and the LN habitats were not balanced, we conducted general linear mixed models (LMMs) to examine the differences in the length of time. In the LMMs, the length of time was treated as the response variable, with the occurrence of bumble bee visitation (categorical factors: present or absent) as the fixed explanatory factor and the site as the random explanatory factor. The length of time was logarithmic transformed to achieve the potential normally distributed residuals and homogeneity of variance. LMMs were conducted using lmerTest package [25], which was built upon the lme4 package [26] to calculate the p value for coefficients using Satterthwate's approximations for degrees of freedom [25]. We ran the anova[kmd] function in the lmerTest package to perform the ANOVA.

### Effects on assemblages of honey bees at the community level

A total of 19 fields from four sites within the NH habitats and 16 fields from another four sites within the LH habitats were selected (Table 1). Within the sites, the number of fields was determined by the availability of squash fields. Mean distance between fields within the site was 115 m. A 35 m × 2 m transect was set in the center of each field. The observers walked along the center line of the transect with a similar speed (approximately five minutes) and recorded the number of flowers with only one honey bee (hereafter one-honey-bee instances), exactly two honey bees (hereafter two-honey-bee instances), three honey bees (hereafter three-honey-bee instances) and four honey bees (hereafter four-honey-bee instances) simultaneously collecting the floral resources on a single flower (S1 Fig). Furthermore, we also counted the number of flowers with one (hereafter one-bumble-bee instances), two, three, and four bumble bees collecting the floral resources. We did not find any flower simultaneously visited by more than four honey bees, more than one bumble bee, or with one honey bee and one bumble bee together on the same flower. The occurrences of one, two, three, and four honey bees on a single flower were identified in this experiment because we aimed to explore the assemblages of honey bees at the community level. The total number of overall honey bees within the transect was achieved by summing the number of honey bees in the four instances. The bumble bees were identified to a genus level in field. After sampling the pollinator abundance, we implemented a random field collection for bumble bees for 10 minutes. Bumble bee specimens were deposited in our laboratory and identified to a species level. Bumble bees contained the most abundant five species *Bombus flavescens*, *B. motivagus*, *B. breviceps*, *B. impetuosus* and *B. avanus*. Ten sample circles with a 0.5 m radius (approximately 0.79 m^2^) separating each other by a distance of 1 m were placed randomly within the transect. Pistillate and staminate flowers in the sample circles were counted and flower sex ratio was calculated as the percentage of pistillate flowers accounting for the total flowers. Each field was surveyed once and carried out between 08:00 am to 10:30 am from May 15 to June 20, 2013.

Distribution patterns of honey bees foraging on squash flowers at the community level were defined here by the percentages of the four instances of honey bees (one, two, three, or four honey bees visiting a single flower) accounting for the overall instances of flowers occupied by honey bees. We used the percentage, instead of the absolute number, of the four instances of honey bees as an indicator to describe the distribution patterns of honey bees on squash flowers because the percentage depicted the dynamics of the four instances of honey bees and revealed their movements between the flowers in field conditions at the community level. For example, behavioral switches or movements of honey bees between flowers, which were potentially driven by bee species interactions, could subsequently lead to variations in the percentages of the four instances of honey bees, but unlikely changed the absolute number of honey bees on the flowers.

Since the percentages of the four instances of honey bees were highly correlated across the 35 fields (S1 Table), we conducted the principal component analysis (PCA) and used the first principal component (PC1) to measure the distribution patterns of honey bees. The two low correlations between the four-honey-bee instances with the two-honey-bee instances and the three-honey-bee instances (S1 Table) unlikely disturbed the results of the PCA because the four-honey-bee instances were only recorded twice in the fields within the LN habitats (see Results) and accounted for less than 0.9% of flower visitations. The PC1 reduced the four variables into a single measure and integrated 96.98% information of original variables. The percentage of the one-honey-bee instances (variable score = -0.82) had a negative loading on PC1, whereas the percentages of two-honey-bee instances (variable score = 0.69), three-honey-bee instances (variable score = 0.12), and four-honey-bee instances (variable score = 0.02) all had positive loadings on PC1. The PC1 score of each field entered into our next analysis. The PCA was conducted using the *vegan* package [27].

We hypothesized that the distribution patterns of honey bees on the squash flowers at the community level were influenced by the following four factors: the number of overall honey bees, the number of overall bumble bees, flower sex ratio, and flower density. A generalized Boosting Regression Trees (BRT) model was applied to explore how the four factors influenced PC1 when traditional modeling techniques, such as general and generalized linear models, could not achieve the normal residuals for our dataset [28]. The BRT modeling technique is widely used in ecology due to its strengths in compatibility for modeling non-linear behavior of ecological processes and tolerance to spatial autocorrelation [29,30]. BRT models were built with the *gbm* package [31] and the *dismo* package [32]. The dismo package supported the extended codes for BRT models written by Elith [30], such as the gbm.step function. The Gaussian loss function was selected in the BRT models because the response variable (PC1) was a continuous variable. Tree complexity was set to 3, with a learning rate of 0.001 and a bag fraction of 0.75, respectively [30,33]. The Individual Variance Inflation Factor (VIF) was less than 4, under the acceptable level of multicollinearity [34]. Goodness-of-fit of models were measured by: (i) the estimated mean cross-validation deviance, (ii) the coefficient of determination (R^2^), and (iii) the Root Mean Squared Error (RMSE) [35,36]. Since flower density and sex ratio explained totally less than 6.3% of PC1 in the full model (four explanatory variables), we removed flower sex ratio and/or flower density from the explanatory variables and refitted the BRT models [30]. However, a reduction in explanatory variables did not increase the RMSE and the coefficient of determination (R^2^), nor did it reduce the estimated mean cross-validation deviance (S2 Table). Therefore, we accepted the full model.

The Wilcoxon test was conducted to test the differences in the percentages of the four instances of honey bees and the number of the four instances of honey bees between the two types of sites. The Pearson correlation coefficients were used to measure the relationship between the number of overall honey bees and the number of overall bumble bees within the sample transect between the two types of sites.

## Results

### Effects on foraging behaviors of honey bees at the individual level

**Fig 2:**
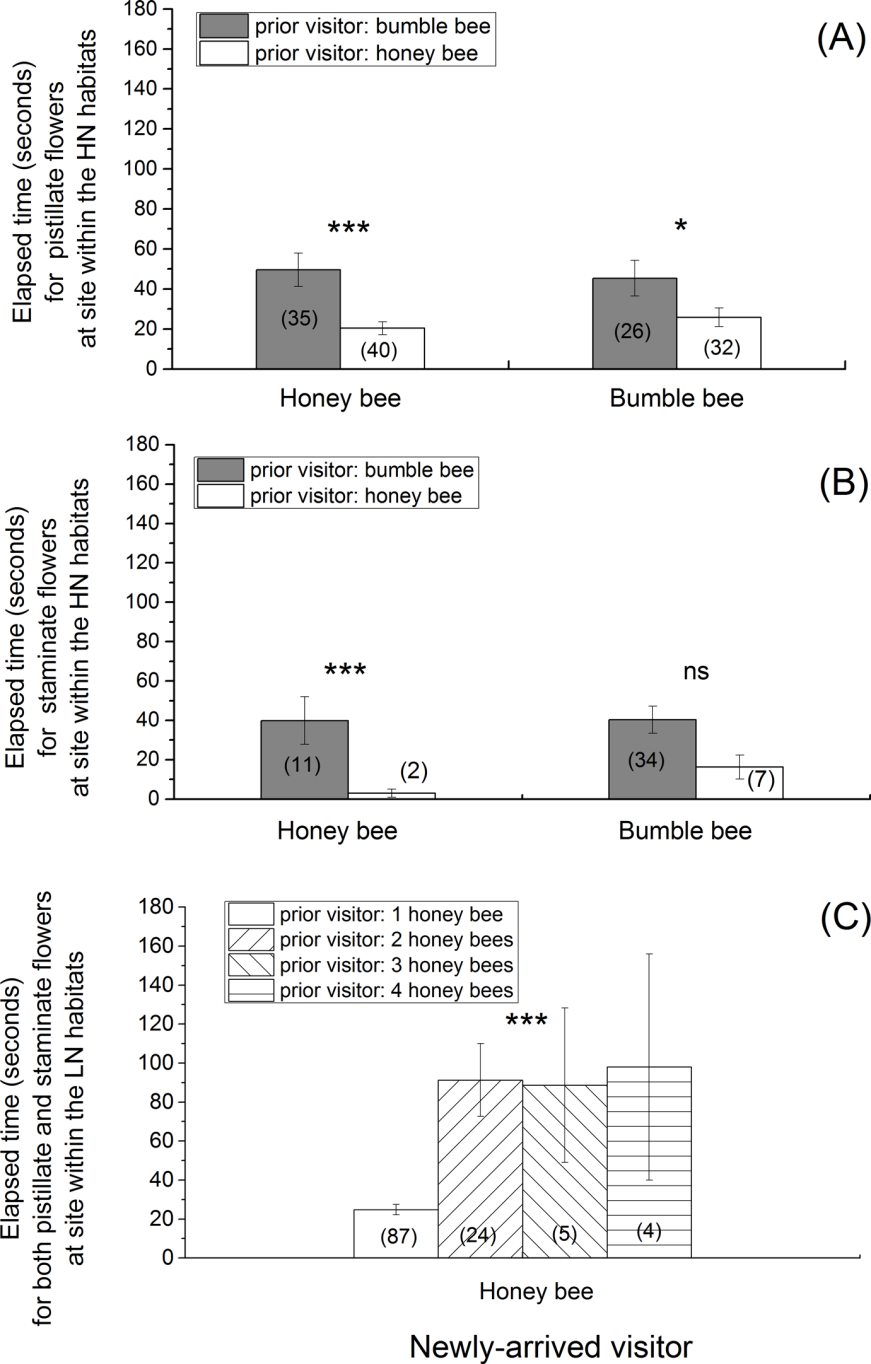
Elapsed time (mean ± se) of the Period for Departure-Arrival Route. (A) pistillate flowers at the fields within the HN habitats; (B) staminate flowers at the fields within the HN habitats; and (C) pistillate and staminate flowers at the fields within the LN habitats. Numbers in parentheses indicate sample size. *** < 0.001; * < 0.05; ns: not significant.

In the fields within the HN habitats, the pistillate flowers previously visited by a bumble bee experienced a significantly longer elapsed time before being revisited again by a new visitor than the flowers previously visited by a honey bee, no matter if the new visitor was a honey bee (Fig 2A left: t = 3.16, *df* = 70.56, *p* = 0.002) or a bumble bee (Fig 2A right: t = 2.07, *df* = 55.65, *p* = 0.04). Similarly, the staminate flowers previously visited by a bumble bee also experienced a significantly longer elapsed time before being revisited by a newly-arrived honey bee than the flowers previously visited by a honey bee (Fig 2B left: t = 3.95, df = 34.51, *p* < 0.001). However, while although the staminate flowers previously visited by a bumble bee experienced a longer time lapse before being revisited by a newly-arrived bumble bee than the flowers previously visited by a honey bee, significant difference was undetectable (Fig 2B right: t = 0.35, df = 20.11, *p* = 0.31). In the fields within the LN habitats, the flowers previously visited by a honey bee required a significantly shorter elapsed time before the arrival of a new honey bee than the flowers previously visited simultaneously by two honey bees, three honey bees, and four honey bees (Fig 2C: ANOVA, F = 18.89, *df* = 3, *p* < 0.001).

**Fig 3:**
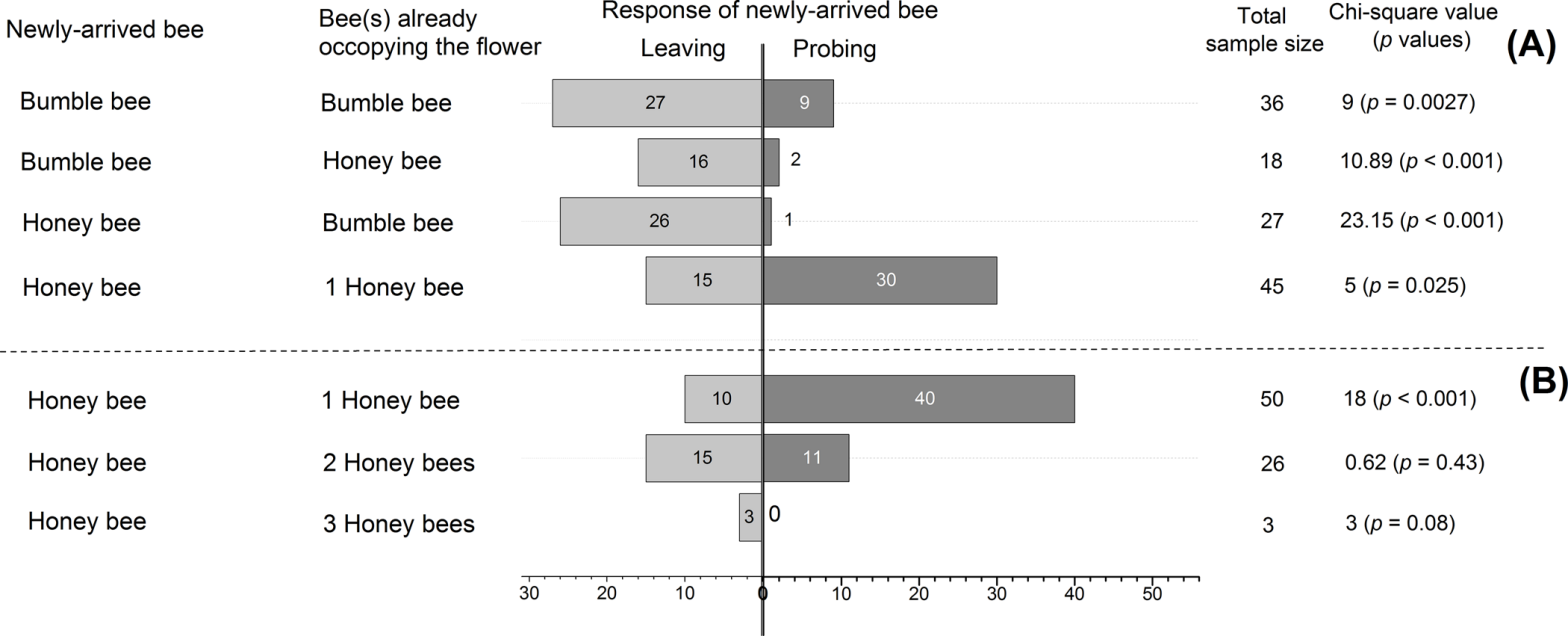
Behavioral responses of a newly-arrived bee to flowers occupied by conspecifics or heterospecifics. (A) responses of a newly-arrived bee in the fields within the HN habitats; (B) responses of a newly-arrived bee in the fields within the LN habitats. Numbers in bars showed the sample size.

In the fields within the HN habitats, if a bumble bee had already occupied the flowers, the newly-arriving visitors, regardless it was a honey bee or a bumble bee, preferred leaving the flowers. Likewise, the newly-arriving bumble bee also preferred leaving the flowers if a honey bee had already occupied the flowers. However, the newly-arriving honey bee preferred probing the flowers if a honey bee was visiting the flowers (Fig 3A). In the fields within the LN habitats, if a honey bee had already occupied the flowers, the newly-arriving honey bee preferred probing the flowers. However, the new-arriving honey bee had an equal probability in response (leaving or probing) to the flowers occupied by two honey bees or three honey bees (Fig 3B).

**Fig 4:**
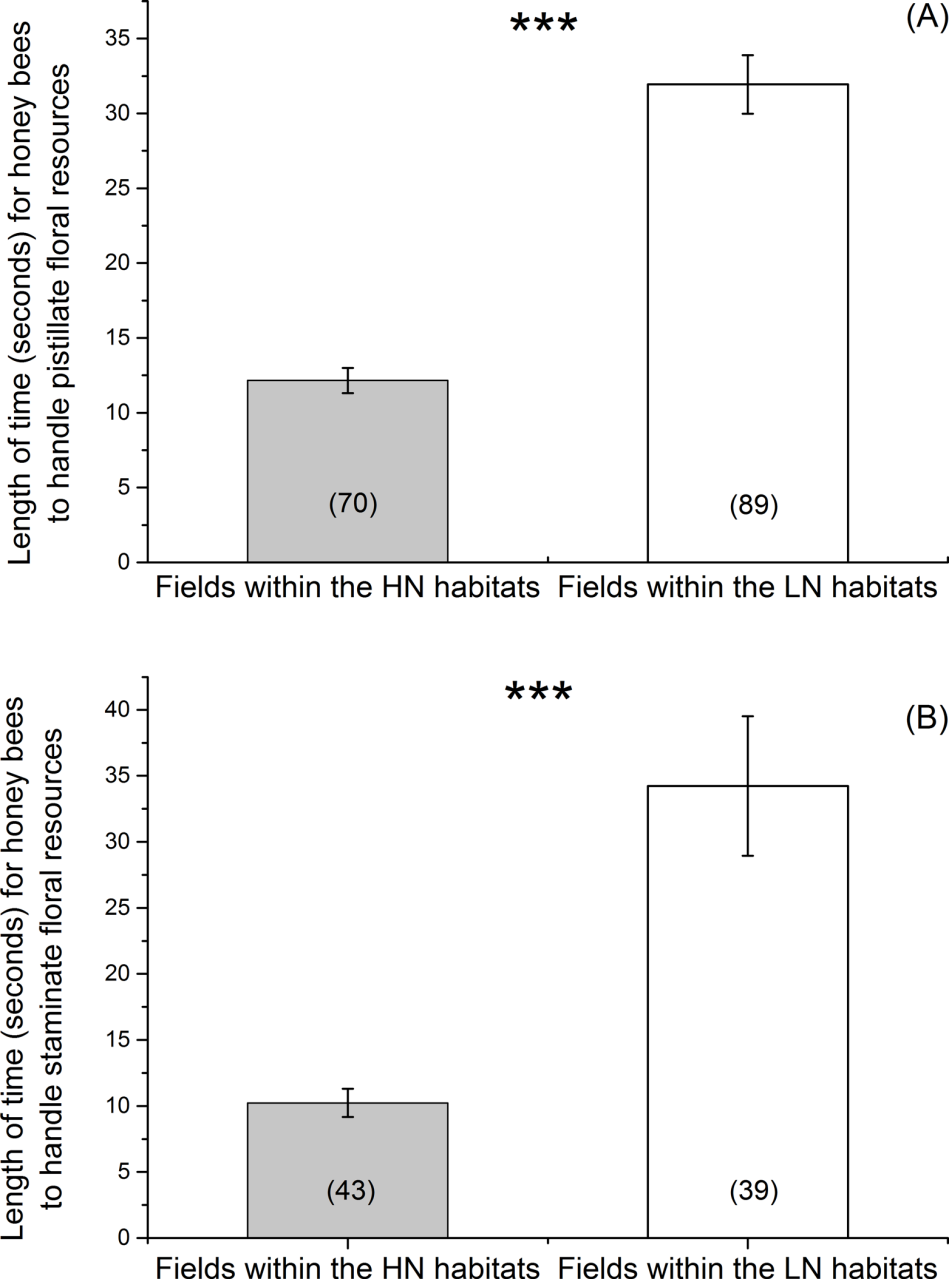
Length of time (mean ± se) used by a honey bee to examine and collect the floral resources. (A) pistillate flowers; (B) staminate flowers. Numbers in parentheses indicated sample size. *** < 0.001.

The honey bees in the fields within the HN habitats spent a significantly shorter amount of time to examine and collect floral resources on pistillate flowers (F = 73.39, *df*_density_ = 4.6, *p* < 0.001) (Fig 4A) and staminate flowers (F = 19.65, *df*_density_ = 3.87, *p* = 0.01) (Fig 4B) than those in the fields within the LN habitats.

### Effects on assemblages of honey bees at the community level

Across the 35 fields within the HN habitats and the LN habitats, 485 one-honey-bee instances, 134 two-honey-bee instances, 24 three-honey-bee instances, and 2 four-honey-bee instances were recorded, which totally corresponded to 833 honey bee visitations. In addition, 110 one–bumble-bee instances were recorded in the fields within the HN habitats. The percentage of one-honey-bee instances was significantly higher in the fields within the HN habitats than in the fields within the LN habitats, whereas the percentages of two-honey-bee instances and three-honey-bee instances were both significantly lower in the fields within the HN habitats than in the fields within the LN habitats. Nevertheless, the difference in the percentage of four-honey-bee instances was not examined between the two types of fields (Table 2).

**Table 2:** Differences in the percentages (median and range) of the four instances of honey bees (one, two, three, and four honey bees visiting a single squash flower) between the HN habitats and the LN habitats.

	Sites within the HN habitats	Sites within the LN habitats	Significance
**One honey bee**	1.00 / 0.82–1.00	0.67 / 0.56–0.76	*p* < 0.001
**Two honey bees**	0.00 / 0.00–0.14	0.28 / 0.13–0.38	*p* < 0.001
**Three honey bees**	0.00 / 0.00–0.04	0.04 / 0.00–0.13	*p* < 0.001
**Four honey bees**	0.00 / 0.00–0.00	0.00 / 0.00–0.04	*p* = 0. 14

**Fig 5:**
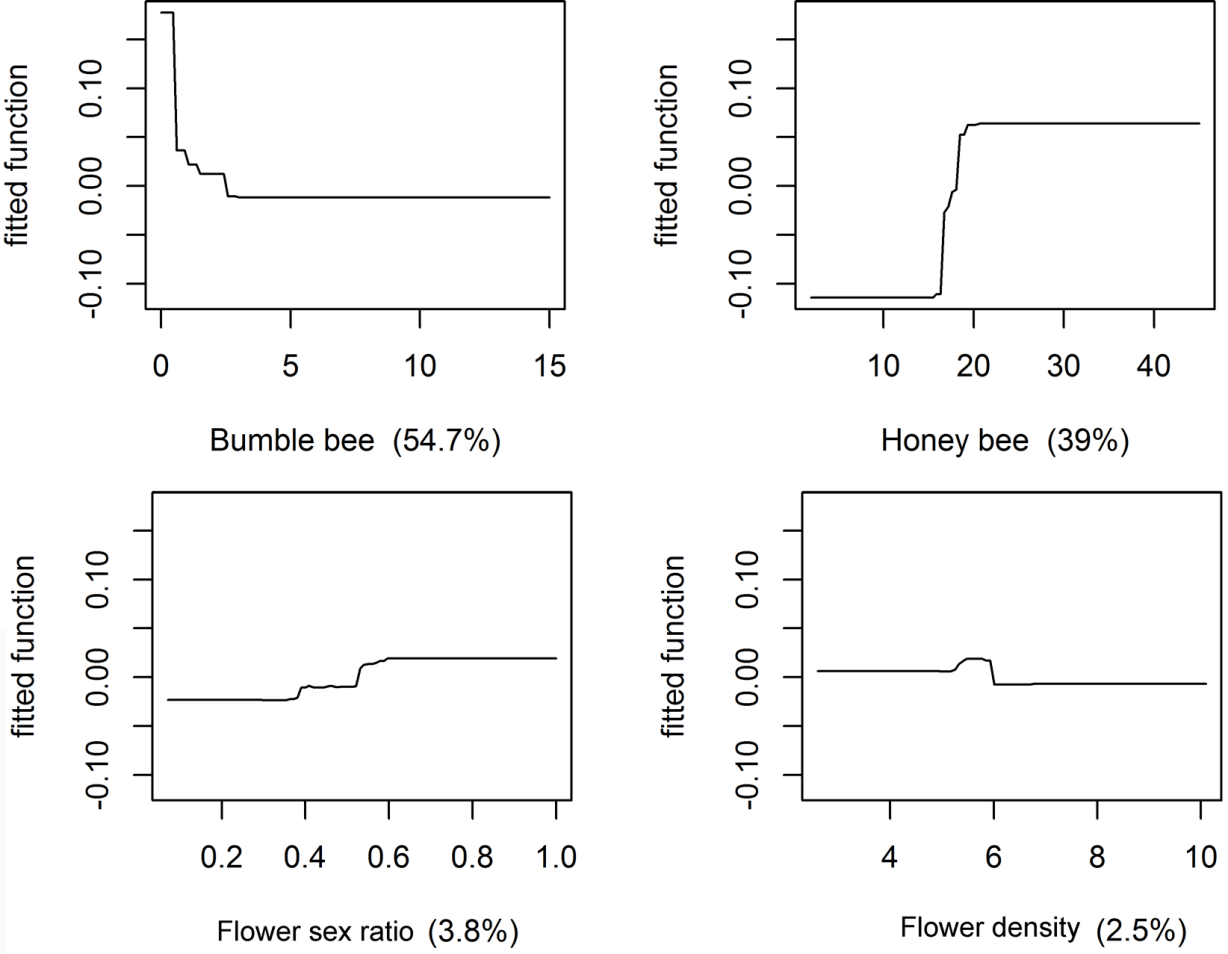
Partial dependence plots for explanatory variables in the BRT model. Y axes were centered on the mean of the response variable. The relative contributions of explanatory variables averaging overall BRT model ensembles are showed as the percentage values in parentheses.

The differences in the distribution patterns of honey bees at the community level (measured by PC1) between the two types of fields were mainly explained in the BRT model by the number of overall bumble bees and the number of overall honey bees (Fig 5; S2 Table). The number of overall bumble bees made the largest contribution to explain the model power (54.7%), followed by a second important contributor, the number of overall honey bees (39.0%). Moreover, the number of overall bumble bees had a negative effect on PC1, whereas the number of overall honey bees had a positive effect on PC1 (Fig 5).

In field conditions, the number of overall honey bees was significantly negatively correlated with the number of overall bumble bees, no matter whether the fields within the HN habitats were analyzed alone (Pearson correlation, r = -0.74; t = -4.35, *df* = 16, *p* < 0.001) or in the context of both types of sites together (Pearson correlation, r = -0.69; t = -5.36, *df* = 32, *p* < 0.001).

Consequently, individually the number of one-honey-bee instances, two-honey-bee instances and three-honey-bee instances were significantly lower in the fields within the HN habitats than in the fields within the LN habitats, but the difference was undetectable for the four-honey-bee instances between the two types of sites. Taken together, the number of instances of overall honey bees (1–4 honey bees) was significantly lower in the fields within the HN habitats than in the fields within the LN habitats (Table 3).

**Table 3:** Differences in the number (median and range) of the four instances of honey bees (one, two, three, and four honey bees visiting a single squash flower) between the HN habitats and the LN habitats.

	Sites within the HN habitats	Sites within the LN habitats	Significance
**One honey bee**	11.5 / 2–23	14 / 11–33	*p =* 0.031
**Two honey bees**	0 / 0–4	7 / 2–14	*p* < 0.001
**Three honey bees**	0 / 0–1	1 / 0–4	*p* < 0.001
**Four honey bees**	0 / 0–0	0 / 0–1	*p =* 0.139
**Total**	12.5 / 2–28	24 / 15–25	*p <* 0.001

## Discussion

Bee species interactions influenced the foraging activities of honey bees on squash flowers in the highland agricultural ecosystems, leading to a longer elapsed time for the flowers to be revisited by a honey bee (Fig 2), an avoidance by the newly-arrived honey bees to visit a flower occupied by a bumble bee (Fig 3), and a short length of time used by honey bees to examine and collect floral resources (Fig 4). Nectar and pollen were two major components for which flower visitors foraged. Different bee species show different capabilities in exploring floral resources [37,38]. The bumble bees had larger body size than honey bees, suggesting they were more effective in exploiting floral resources [18]. After finishing visiting the squash flowers, bumble bees likely left only a little amount of floral resources on the squash flowers, which reduced the attractiveness of the flowers to the new visitors. Moreover, it has been evidenced that flower visitors secrete chemical components on flowers [39], which can be detected by conspecifics and heterospecifics [40]. Pollinators show different responses (avoidance or acceptance) to the cues [11,41]. The flower visitors (e.g. honey bees and bumble bees) in the squash agro-ecosystem also may use the cues to find the flowers. The chemical components from bumble bees were likely deterrents to honey bees, resulting in the avoidance by honey bees to visit a flower previously visited by a bumble bee. The chemical components left behind in a flower from honey bees were likely detrimental to bumble bees as well, but neutral to honey bees. The two mechanisms could explain why bumble bee visitations caused an increase in the amount of elapsed time before flowers were visited by a honey bee. Furthermore, species recognition during flower visitation might occur and alter the foraging behavior of bees [12,16], propelling them to move between flowers [8,42] or among crop rows [7]. Our field observations were consistent with those studies. Honey bees avoided the flowers occupied by bumble bees, but accepted the flowers occupied by honey bees.

The effect of specific interactions at the individual level can be amplified and further transferred to all pollinators at the community level, causing a change in the assemblages of honey bees on squash flowers (Table 3). This mechanism by which bee interactions influenced the number of pollinators on squash flowers was different from those already comparatively well understood [43], which found that decline in pollinator visitations was derived from a loss of pollinator populations in habitats [8,44,45]. However, in the highland agricultural ecosystems, a decline in the number of honey bees on squash flowers in the HN habitats was explained by bee species interactions, not by the change in the local honey bee populations. Indeed, bee interactions occur when two individuals forage for the same floral resources [16]. Noticeably, its intensity and direction should be specified to bee species and their abundances, with a high likelihood of occurrence in situations where one species over-exploits the floral resources. Therefore, a decline in the number of honey bees on squash flowers should only be treated as a short-term behavioral response to bumble bees co-foraging for the same floral resources.

Bee interactions have a critical role in ecosystem functioning. Honey bees and bumble bees are two functional groups in pollinating the squash flowers in the highland agricultural ecosystems, with bumble bees being more effective in transferring and depositing pollen than honey bees [18]. Our experiments found an increase in the number of honey bees on squash flowers in the fields that had a low occurrence of wild bumble bees (Table 3). Although the squash flowers in the LN habitats experienced a decline in bumble bees (Table 1), they had an increase in the number of overall honey bees on flowers, thus providing an opportunity to enhance the flower visitation rate of honey bees. From this point, bee interactions could enhance, to some level, the resilience of pollination services of agricultural ecosystems against biodiversity loss. It is also important, however, to notice that the amount of overall pollen deposited on a stigma surface (pollination services) is related to a pollinator’s efficacy (e.g. single visit deposition of pollen grains) [18,46]. An increase in the number of honey bees in the fields does not necessarily stand for an improvement of overall pollen gains on stigmas.

Deliberate importation of managed honey bees into agricultural ecosystems often improves crop pollination when wild pollinators are insufficient [47,48]. The effectiveness of those practices needs to consider the spatial variation of wild pollinators (also feral honey bees) within the landscapes. The number of honey bees in the HN habitats was negatively associated with the number of bumble bees. Therefore, placing additional managed honey bee colonies in those fields likely would lead to resource competitions and force some bees to select different floral resources. Economic benefits could be achieved only when honey bee colonies are placed where wild bees are already very sparse and discrete. Similarly, the farming practices which introduce floral and nesting resources into ecosystems to restore and rebuild wild bee populations also need to consider the feral and managed honey bees already in the landscapes.

## Supporting Information

S1 Fig Four instances of honey bees (one, two, three and four honey bees visiting a single squash flower) in the LN habitats. One honey bee (A), two honey bees (B), three honey bees (C) and four honey bees (D).
(TIF)

S1 Table Pearson correlation coefficients among the percentages of the four instances of honey bees (one, two, three, and four honey bees visiting a single squash flower). *** < 0.001; ** < 0.01; * < 0.05; ns: not significant.
(DOC)

S2 Table Characteristics of the full and simplified BRT models ^a.^ (DOC)
