# Supplementary figures and images for: The Potential Influence of Bumble Bee Visitation on Foraging Behaviors and Assemblages of Honey Bees on Squash Flowers in Highland Agricultural Ecosystems
Source: PLoS One. 2016 Jan 14;11(1):e0144590. doi: 10.1371/journal.pone.0144590 (PMC4713089; doi:10.1371/journal.pone.0144590)

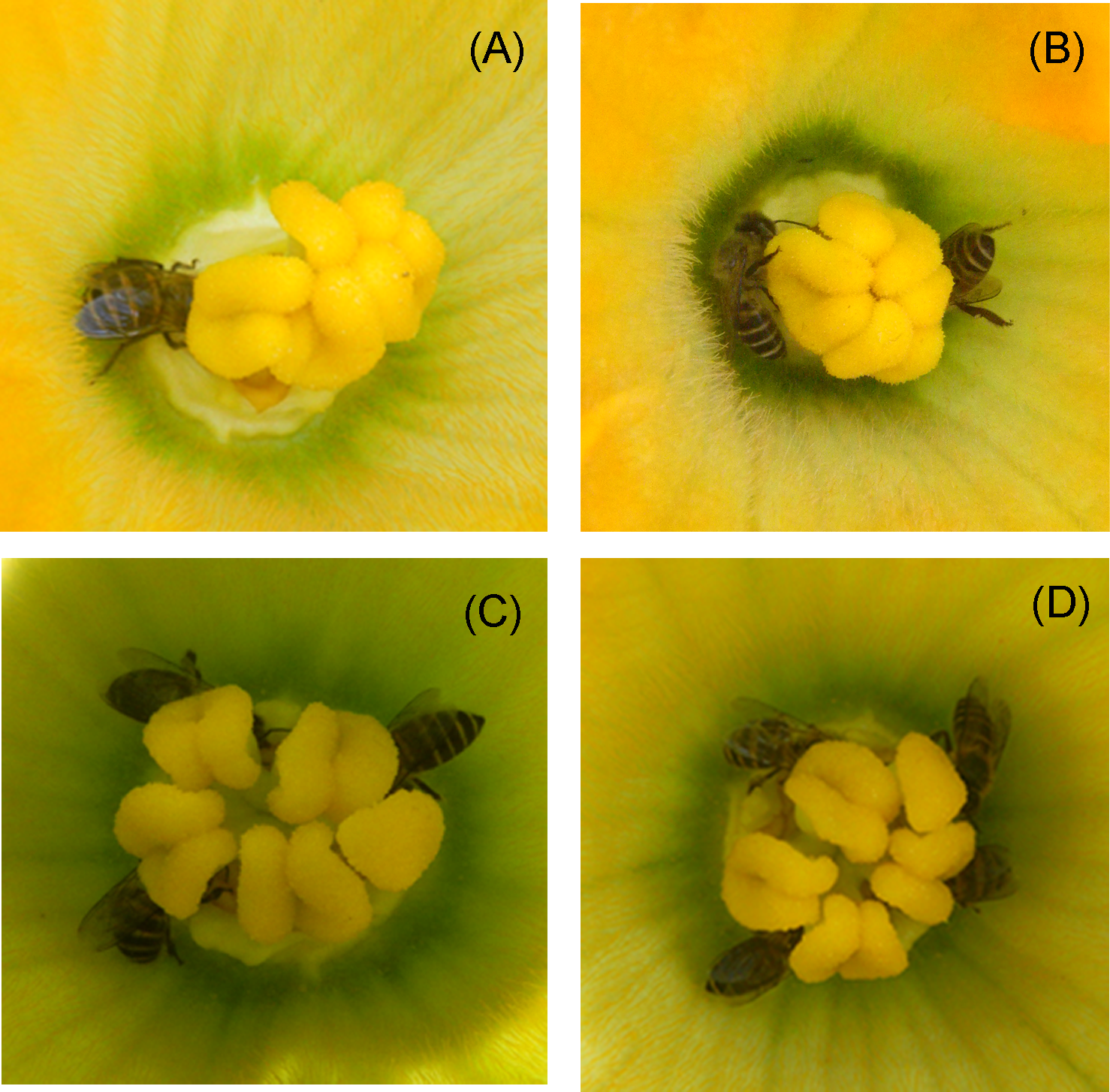

Supplement: S1 Fig — One honey bee (A), two honey bees (B), three honey bees (C) and four honey bees (D). (TIF) [file pone.0144590.s001.tif]
