# Supplementary material for: The Potential Influence of Bumble Bee Visitation on Foraging Behaviors and Assemblages of Honey Bees on Squash Flowers in Highland Agricultural Ecosystems
Source: PLoS One. 2016 Jan 14;11(1):e0144590. doi: 10.1371/journal.pone.0144590 (PMC4713089; doi:10.1371/journal.pone.0144590)
Supplement: S1 Table — *** < 0.001; ** < 0.01; * < 0.05; ns: not significant. (DOC) [file pone.0144590.s002.doc]

**Table S1. Pearson correlation coefficients among the percentages of the four instances of honey bees (one, two, three, and four honey bees visiting a single squash flower).**

***** < 0.001; ** < 0.01; * < 0.05; ns: not significant.**

|  | Two honey bees | Three honey bees | Four honey bees |
| --- | --- | --- | --- |
| One honey bee | - 0.977 *** | - 0.696 *** | - 0.389 * |
| Two honey bees |  | 0.534 *** | 0.327 ns |
| Three honey bees |  |  | 0.270 ns |

*** < 0.001; ** < 0.01; * < 0.05; ns: not significant.
