# Supplementary material for: The Potential Influence of Bumble Bee Visitation on Foraging Behaviors and Assemblages of Honey Bees on Squash Flowers in Highland Agricultural Ecosystems
Source: PLoS One. 2016 Jan 14;11(1):e0144590. doi: 10.1371/journal.pone.0144590 (PMC4713089; doi:10.1371/journal.pone.0144590)
Supplement: S2 Table — (DOC) [file pone.0144590.s003.doc]

**Table S2. Characteristics of the full and simplified BRT models** ^a^

| **Characteristics** | **Full model** | **Simplified model1** | **Simplified model2** |
| --- | --- | --- | --- |
| **No. sites** | 34 | 34 | 34 |
| **No. trees** | 5150 | 5050 | 4150 |
| **No. explanatory variables** | 4 | 3 | 2 |
| **Bumble bee’s contribution** | 54.7 % | 57.5 % | 59.20 % |
| **Honey bee’s contribution** | 39.0 % | 39.2 % | 40.80 % |
| **Flower density’s contribution** | 3.8 % | 3.3 % | —— |
| **Flower sex ratio’s contribution** | 2.5 % | —— | —— |
| **Mean total deviance ± se** | 0.036 ± 0.005 | 0.036 ± 0.005 | 0.036 ± 0.005 |
| **CV correlation** | 0.927 | 0.925 | 0.924 |
| **Training data correlation** | 0.935 | 0.929 | 0.922 |
| **Estimated cross-validation deviance ± se** | 0.008 ± 0.002 | 0.008 ± 0.002 | 0.008 ± 0.002 |
| **RMSE** | 0.066 | 0.069 | 0.072 |
| **R^2^** | 0.874 | 0.863 | 0.854 |

^a^ Full model used honey bee abundance, bumble bee abundance, flower density and flower sex ratio as explanatory variables, whereas simplified model 1 excluded flower sex ratio and simplified model 2 excluded flower sex ratio and flower density. Goodness-of-fit was measured by estimated cross-validation deviance, RMSE and R^2^. Low cross-validation deviance, RMSE and high R^2^ support a good model (Dawson, 2007).

Dawson *et al.*, 2007. HydroTest, A web-based toolbox of evaluation metrics for the standardised assessment of hydrological forecasts. Environmental Modelling & Software, 22, 1034-1052
